# Stimulated myoblast differentiation on graphene oxide-impregnated PLGA-collagen hybrid fibre matrices

**DOI:** 10.1186/s12951-015-0081-9

**Published:** 2015-03-12

**Authors:** Yong Cheol Shin, Jong Ho Lee, Linhua Jin, Min Jeong Kim, Yong-Joo Kim, Jung Keun Hyun, Tae-Gon Jung, Suck Won Hong, Dong-Wook Han

**Affiliations:** Department of Cogno-Mechatronics Engineering & BK21+ Nano-Integrated Cogno-Mechatronics Engineering, Pusan National University, Busan, 609-735 South Korea; Department of Biosystems Machinery Engineering, Chungnam National University, Daejeon, 305-764 South Korea; Department of Rehabilitation Medicine, College of Medicine, Dankook University, Cheonan, 330-714 South Korea; Department of Nanobiomedical Science, BK21PLUS NBM Global Research Center, Dankook University, Cheonan, 330-714 South Korea; Institute of Tissue Regeneration Engineering, Dankook University, Cheonan, 330-714 South Korea; Osong Medical Innovation Foundation, Medical Device Development Center, Cheongju, 363-951 South Korea

**Keywords:** Electrospun fibre matrix, Poly(lactic-co-glycolic acid), Graphene oxide, Collagen, Myoblast differentiation, Skeletal tissue engineering

## Abstract

**Background:**

Electrospinning is a simple and effective method for fabricating micro- and nanofiber matrices. Electrospun fibre matrices have numerous advantages for use as tissue engineering scaffolds, such as high surface area-to-volume ratio, mass production capability and structural similarity to the natural extracellular matrix (ECM). Therefore, electrospun matrices, which are composed of biocompatible polymers and various biomaterials, have been developed as biomimetic scaffolds for the tissue engineering applications. In particular, graphene oxide (GO) has recently been considered as a novel biomaterial for skeletal muscle regeneration because it can promote the growth and differentiation of myoblasts. Therefore, the aim of the present study was to fabricate the hybrid fibre matrices that stimulate myoblasts differentiation for skeletal muscle regeneration.

**Results:**

Hybrid fibre matrices composed of poly(lactic-co-glycolic acid, PLGA) and collagen (Col) impregnated with GO (GO-PLGA-Col) were successfully fabricated using an electrospinning process. Our results indicated that the GO-PLGA-Col hybrid matrices were comprised of randomly-oriented continuous fibres with a three-dimensional non-woven porous structure. Compositional analysis showed that GO was dispersed uniformly throughout the GO-PLGA-Col matrices. In addition, the hydrophilicity of the fabricated matrices was significantly increased by blending with a small amount of Col and GO. The attachment and proliferation of the C2C12 skeletal myoblasts were significantly enhanced on the GO-PLGA-Col hybrid matrices. Furthermore, the GO-PLGA-Col matrices stimulated the myogenic differentiation of C2C12 skeletal myoblasts, which was enhanced further under the culture conditions of the differentiation media.

**Conclusions:**

Taking our findings into consideration, it is suggested that the GO-PLGA-Col hybrid fibre matrices can be exploited as potential biomimetic scaffolds for skeletal tissue engineering and regeneration because these GO-impregnated hybrid matrices have potent effects on the induction of spontaneous myogenesis and exhibit superior bioactivity and biocompatibility.

## Background

Recently, many studies have focused on the fabrication of artificial scaffolds as potential biomimetic substrates, which can control the cellular behaviours and provide appropriate microenvironments. Ideal scaffolds should have a suitable structure for the growth of cells because they interact directly with the cells. In addition, these scaffolds should have biofunctionality because the cell fate is controlled by various mechanical and biochemical cues from the surrounding environment. Therefore, over the past few years considerable efforts have been made to meet these requisites using a variety of methods and materials. Several techniques have been used to fabricate artificial scaffolds that mimic the natural extracellular matrix (ECM) such as electrospinning, self-assembly, phase separation, and drop casting [1,2]. Among these techniques, electrospinning has attracted numerous interests as a simple and effective method [3]. Many studies have reported that artificial scaffolds composed of biocompatible polymers and various biomaterials can be manufactured by electrospinning [3-8]. In particular, electrospun matrices, composed of various polymers, including polyesterurethane, poly(ε-caprolactone) and poly(hydroxybutyrate), coated with proteins or not, have been designed as potential artificial scaffolds for skeletal muscle regeneration [9-12].

Over the last decade, poly(lactic-*co*-glycolic acid, PLGA), collagen (Col) and/or their combination have been used extensively as artificial scaffolds [13-16]. Col is a major component of the ECM and has been widely used as a representative biomaterial for the tissue engineering applications because of its many advantages, including excellent biocompatibility, cell affinity and bioresorbability. On the other hand, the poor mechanical and rapidly degrading properties of Col remain obstacles to its use as a scaffold for tissue engineering. PLGA is a biocompatible polymer that is extensively used for a range of biomedical applications, such as surgical suturing, drug delivery carriers, and tissue engineering scaffolds because of its outstanding features, such as biocompatibility, biodegradability, good solubility in organic solvents, and suitable mechanical properties for scaffolds [17-19].

Graphene, a single two-dimensional layer of carbon, and its derivatives have attracted considerable attention for biomedical applications, such as scaffold substrates, drug or gene delivery carriers, and bio-sensors [20-23]. Graphene oxide (GO) is the oxidised form of graphene and has many oxygen-containing functional moieties, such as hydroxyl, carboxyl and epoxy groups. Recently, it was reported that GO has good biocompatibility and can enhance both the mechanical properties of the substrates and the cellular behaviours [24-27]. Therefore, GO might have potential use as a bio-building block for scaffold substrates. In the present study, GO-impregnated biomimetic matrices composed of PLGA and Col (GO-PLGA-Col), which are analogous to the natural ECM, were fabricated via an electrospinning process. The use of PLGA-Col in combination with GO is novel and challenging. The hypothesis tested was whether these GO-PLGA-Col hybrid fibre matrices would be beneficial to myoblast differentiation. For this purpose, the physicochemical and mechanical properties of the matrices were characterised by field emission scanning electron microscopy (FESEM), atomic force microscopy (AFM), Fourier transform-infrared (FT-IR) spectroscopy, contact angle measurements, X-ray diffraction (XRD), and tensile tests. Furthermore, the cellular behaviours of C2C12 skeletal myoblasts cultured on GO-PLGA-Col hybrid matrices were examined to explore their biocompatibility and bioactivity.

## Results and discussion

### Physicochemical and mechanical characteristics of GO-PLGA-Col fibre matrices

**Figure 1:**
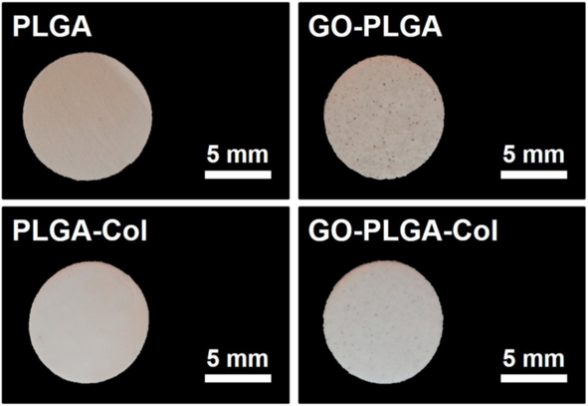
**Digital photographs of the PLGA, GO-PLGA, PLGA-Col, and GO-PLGA-Col fibre matrices.** All photographs shown in this figure are representative of six samples with similar results.

**Figure 2:**
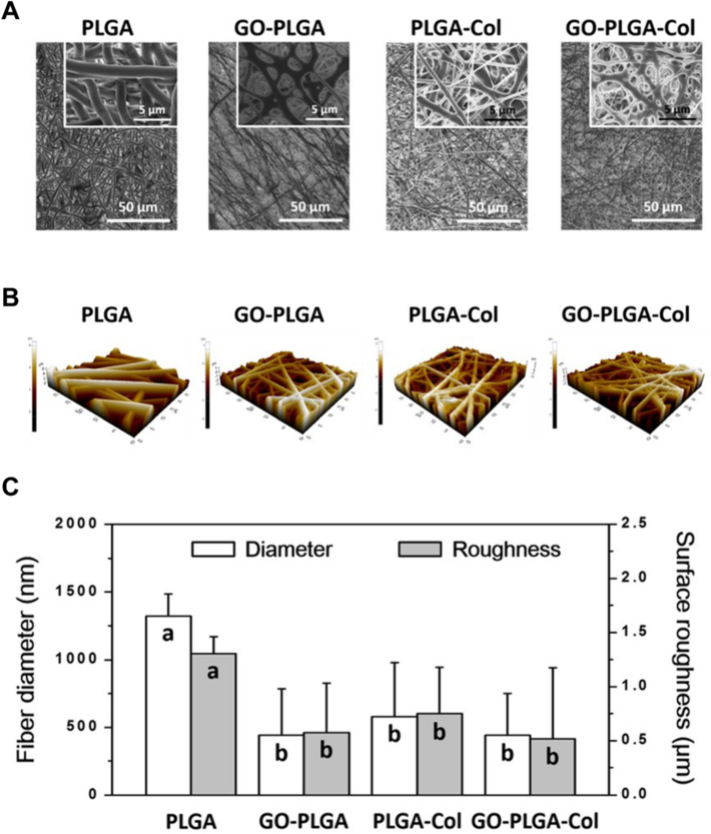
**Surface morphological and topographical images of PLGA, GO-PLGA, PLGA-Col, and GO-PLGA-Col fibre matrices. (A)** FESEM images and **(B)** AFM images of PLGA, GO-PLGA, PLGA-Col, and GO-PLGA-Col fibre matrices. All photographs shown in this figure are representative of six independent experiments with similar results. **(C)** Correlation between fibre diameter of matrices and their surface roughness (*R*_a_). The different letters in **(C)** denote the significant differences between each experimental group, *p* < 0.05. If two groups have the same single letter (a, b), there is no significant difference between them.

GO-PLGA-Col hybrid fibre matrices were prepared by electrospinning the admixture of PLGA and Col blended with GO. The matrices contained a large number of black spots scattered throughout the matrix due to impregnated GO (Figure 1). The physicochemical properties of the GO-PLGA-Col hybrid matrices were characterised by FESEM, AFM and FT-IR spectroscopy. As shown in Figure 2A, FESEM images showed that all matrices had a three-dimensional network structure with interconnected pores similar to that of the natural ECM. The diameter of the GO-PLGA-Col fibres showed a wide range between 100 and 950 nm with the average diameter of 440 nm due to the random alternation of Col and PLGA as well as GO impregnation. The average diameter of the hybrid fibres substantially decreased when GO, Col or both were blended with PLGA. A series of evidence supports this result, showing that the average diameter of the PLGA fibres blended with GO or Col is much smaller than that of pure PLGA fibres [28,29]. This phenomenon can be explained partly by the fact that the viscosity of the electrospinning solution was decreased due to the blending of GO and/or Col [30]. The surface topography of each fibre matrix correlated well with its own FESEM image (Figure 2B). The average surface roughness (*R*_a_) of PLGA, GO-PLGA, PLGA-Col, and GO-PLGA-Col fibre matrices was 1.30, 0.58, 0.75, and 0.52 μm, respectively. The surface roughness of the matrices was closely related to the fibre diameter (Figure 2C). Some previous studies have revealed that the surface roughness of the fibrous matrices decreases with decreasing diameter of the constituent fibre [31,32]. Furthermore, there is a general tendency that the surface-area-to-volume ratio of matrices increases with decreasing fibre diameter [33]. Therefore, it is suggested that GO-PLGA-Col matrices would interact easily with the cells due to their increased surface-area-to-volume ratio.

**Figure 3:**
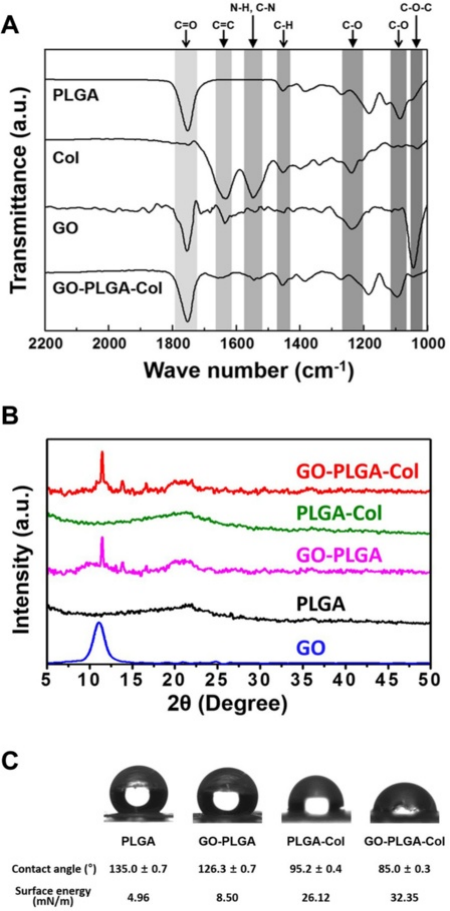
**Physicochemical characterization of the PLGA, GO-PLGA, PLGA-Col, and GO-PLGA-Col matrices. (A)** FT-IR spectra were recorded in absorption mode in the wavelength range of 1000–2200 cm^−1^ with a resolution of 4.0 cm^−1^ and 16-times scanning. **(B)** XRD patterns of GO, PLGA, GO-PLGA, PLGA-Col, and GO-PLGA-Col matrices were collected with Cu-K_α_ radiation (λ = 0.154 nm) at 40 kV and 30 mA. **(C)** Contact angles of the matrices were measured by the sessile drop method.

Figure 3A shows the FT-IR spectra of PLGA, GO-PLGA, PLGA-Col, and GO-PLGA-Col fibres. In the GO-PLGA-Col fibres, a noticeable band was observed at 1752 cm^−1^, which was assigned to the C = O stretching vibration. This band was derived from PLGA and GO [34]. The characteristic band for Col was observed at 1546 cm^−1^, which can be attributed to the amide II vibration from the N-H bending and C-N stretching vibrations [35]. On the other hand, another band at 1632 cm^−1^ was observed, which was assigned to C = C bonds related to the unoxidised sp^2^ carbon of GO [36,37]. This result implied that GO and Col were well distributed in the GO-PLGA-Col fibre matrices. The XRD patterns of GO and electrospun matrices are presented in Figure 3B. XRD pattern of GO showed a strong diffraction peak at 11.09°, corresponding to the interlayer spacing of 0.797 nm calculated by using the Bragg equation [38]. In contrast, PLGA and PLGA-Col matrices did not show any diffraction peak due to their amorphous structure. On the other hand, the XRD patterns of GO-PLGA and GO-PLGA-Col matrices exhibited a sharp diffraction peak at 11.46°, corresponding to the interlayer spacing of 0.772 nm. This result indicated that the GO maintained the structure of a single two-dimensional layer of carbon with oxygen-containing functional moieties within the GO-PLGA or GO-PLGA-Col matrices. Figure 3C shows the water contact angles and surface energies of the PLGA, GO-PLGA, PLGA-Col, and GO-PLGA-Col matrices. The hydrophilicity of the matrices plays an important role in interacting with cells. The water contact angle was 135.0 ± 0.7° for the PLGA matrices, 126.3 ± 0.7° for the GO-PLGA matrices, 95.2 ± 0.4° for the PLGA-Col matrices, and 85.0 ± 0.3° for the GO-PLGA-Col matrices. The water contact angles of the GO-PLGA matrices were slightly decreased by blending with GO because of not only the hydroxyl groups but also the negatively charged groups, such as carboxylic acid groups on the GO surface [25]. On the other hand, the water contact angles of the matrices were significantly (*p* < 0.05) decreased by blending with Col because of its hydrophilicity. Among the matrices, the lowest contact angle was obtained for the GO-PLGA-Col matrices. In addition, the surface energy of GO-PLGA-Col matrices was measured to be 32.35 mN/m, which was much higher than that of the PLGA matrices (4.96 mN/m). These results suggest that the GO-PLGA-Col matrices have a more hydrophilic surface compared to that of the other matrices. The improvement in the hydrophilicity of the scaffold surface enhances the cellular behaviours, including the initial attachment, proliferation and differentiation [39,40]. Therefore, GO-PLGA-Col matrices can provide a suitable microenvironment for the attachment and proliferation of cells because they have a more hydrophilic surface than the other matrices.

**Figure 4:**
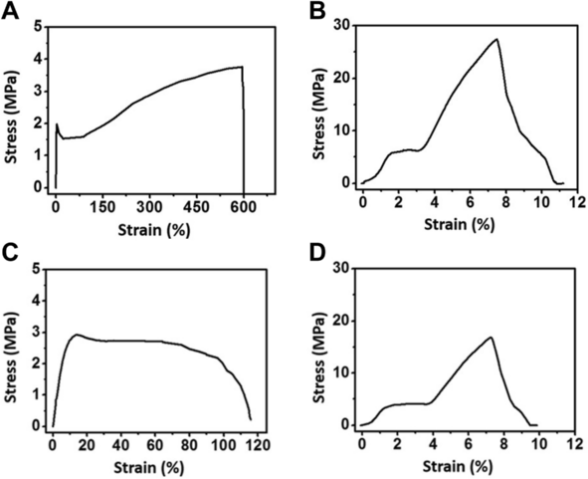
**Stress–strain curves of the matrices.** Stress–strain curves of the **(A)** PLGA, **(B)** GO-PLGA, **(C)** PLGA-Col, and **(D)** GO-PLGA-Col matrices were obtained under a cross-head speed of 10 mm/min. Prior to testing, 4 types of matrices were cut into a rectangular shape, 40 mm in length and 10 mm in width.

Figure 4 shows the stress–strain curves of the matrices under a tensile load. The tensile strength and elastic modulus of the matrices can be obtained from the stress–strain curves. The tensile strengths of the PLGA, GO-PLGA, PLGA-Col, and GO-PLGA-Col matrices were approximately 3.8, 27.4, 2.9, and 16.8 MPa, respectively. The elastic moduli of the PLGA, GO-PLGA, PLGA-Col, and GO-PLGA-Col matrices were approximately 140, 690, 50, and 460 MPa, respectively. The elastic moduli were taken as the maximum linear slope of the stress–strain curve in the elastic deformation region [16,41]. When Col was added to the polymer substrates, the mechanical properties of the matrices, such as the tensile strength and elastic modulus, decreased [28,42]. Therefore, the tensile strength and elastic modulus of the matrices were decreased by blending with Col. In contrast, the tensile strength and elastic modulus of the GO-impregnated matrices were remarkably higher than those of the PLGA and PLGA-Col matrices. Previous studies reported that the mechanical properties of substrates were improved by the addition of GO because of the interfacial interaction between the oxygen-containing functional moieties of GO and the hydroxyl or amine groups of the substrates [25,26,43,44]. Therefore, this result suggests that the poor mechanical properties of Col-contained matrices can be reinforced by the impregnation of GO, and the GO-PLGA-Col matrices can serve as mechanically stable scaffolds for cell growth.

### Attachment and proliferation of C2C12 skeletal myoblasts on GO-PLGA-Col fibre matrices

**Figure 5:**
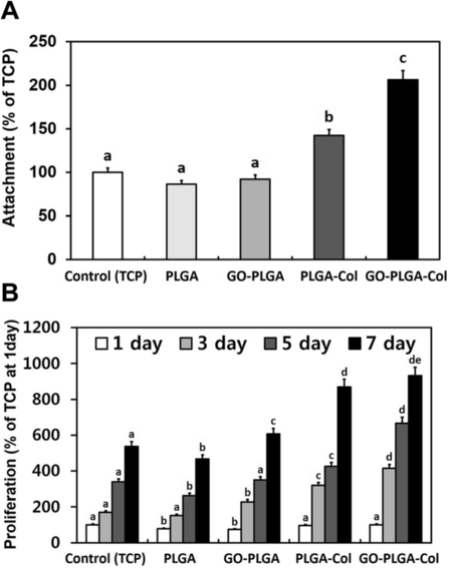
**Initial attachment and proliferation of C2C12 skeletal myoblasts. (A)** Initial attachment of C2C12 skeletal myoblasts on tissue culture plastic (TCP), PLGA matrices, GO-PLGA matrices, PLGA-Col matrices, and GO-PLGA-Col matrices were measured using a CCK-8 assay at 6 hours after seeding. **(B)** Proliferation of C2C12 skeletal myoblasts were measured using CCK-8 assay on 1, 3, 5, and 7 days after incubation. The different letters in **(A)** denote the significant differences between the control and experimental groups, *p* < 0.05. The different letters in **(B)** denote the significant differences between the control and experimental groups at the same time point, *p* < 0.05. If two groups have the same single letter (a, b, c, etc.), there is no significant difference between them. If a group is marked with a dual letter (eg, de), it has a significant difference from the control and other groups marked with ‘a’, ‘b’, or ‘c’, but does not from another group marked with ‘d’.

C2C12 skeletal myoblasts were cultured on the fibre matrices to evaluate the cellular behaviours including the initial attachment and proliferation on GO-PLGA-Col fibre matrices. C2C12 skeletal myoblasts were seeded on all matrices and the cell viability was measured at 6 hours after seeding. The initial attachment of the C2C12 skeletal myoblasts cultured on the PLGA-Col and GO-PLGA-Col matrices was significantly (*p* < 0.05) higher than that on the tissue culture plastic (TCP) control, PLGA matrices and GO-PLGA matrices, as shown in Figure 5A and Table 1. This suggests that the initial attachment of cells is influenced greatly by the hydrophilic/hydrophobic properties of the substrate surface. In addition, proliferation of cells on the matrices on 1, 3, 5, and 7 days after incubation was evaluated (Figure 5B and Table 1). The proliferation of cells was consistently increased during the culture period in all groups. On the other hand, the proliferation of cells on the GO-PLGA-Col matrices was significantly (*p* < 0.05) better than that on the other groups. This might be due to the improved hydrophilicity of the matrices and the ability of GO and Col to support proliferation of cells. Previous studies demonstrated that GO enhances the cellular behaviours, including attachment, proliferation, and even differentiation [24-26,45]. Moreover, it has been well known that Col supports cell attachment and proliferation because of its outstanding biocompatibility and cell affinity [28,29,42]. Many studies have reported that an improvement in attachment or proliferation of myoblasts promotes their myogenic differentiation due to the community effect where cells can undergo myogenic differentiation when they reach confluence for contact of the neighbouring cells [46-52]. Therefore, Col enables not only to enhance attachment and proliferation but also to help myogenic differentiation. It is suggested that the GO-PLGA-Col matrices can effectively promote the initial attachment and proliferation of cells by GO and Col.

**Table 1:** **Initial attachment and proliferation of C2C12 skeletal myoblasts on tissue culture plastic (TCP) and electrospun matrices**

	^**(a)**^ **Attachment at 6 hours (%)**	^**(b)**^ **Proliferation at 1 day (%)**	^**(b)**^ **Proliferation at 3 days (%)**	^**(b)**^ **Proliferation at 5 days (%)**	^**(b)**^ **Proliferation at 7 days (%)**
Control (TCP)	100	100	170.47 ± 2.23	339.18 ± 8.60	537.24 ± 2.63
PLGA	86.14 ± 0.88	78.71 ± 0.86	152.51 ± 3.45	262.40 ± 15.62	467.23 ± 18.68
GO-PLGA	92.14 ± 2.30	74.25 ± 0.89	228.11 ± 3.90	350.38 ± 14.20	607.72 ± 7.21
PLGA-Col	142.18 ± 1.90	96.69 ± 0.62	319.83 ± 5.35	426.23 ± 3.12	869.55 ± 8.96
GO-PLGA-Col	206.36 ± 1.43	100.55 ± 0.29	415.34 ± 3.57	666.7 ± 24.28	932.89 ± 24.28

(a) The values mean the percentage ratio of the cell viability on PLGA, GO-PLGA, PLGA-Col, and GO-PLGA-Col matrices to that on TCP. (b) The values mean the percentage ratio of the cell viability on PLGA, GO-PLGA, PLGA-Col, and GO-PLGA-Col matrices at each time point to that on TCP at initial time point (1 day).

### Myogenic differentiation of C2C12 skeletal myoblasts on GO-PLGA-Col fibre matrices

**Figure 6:**
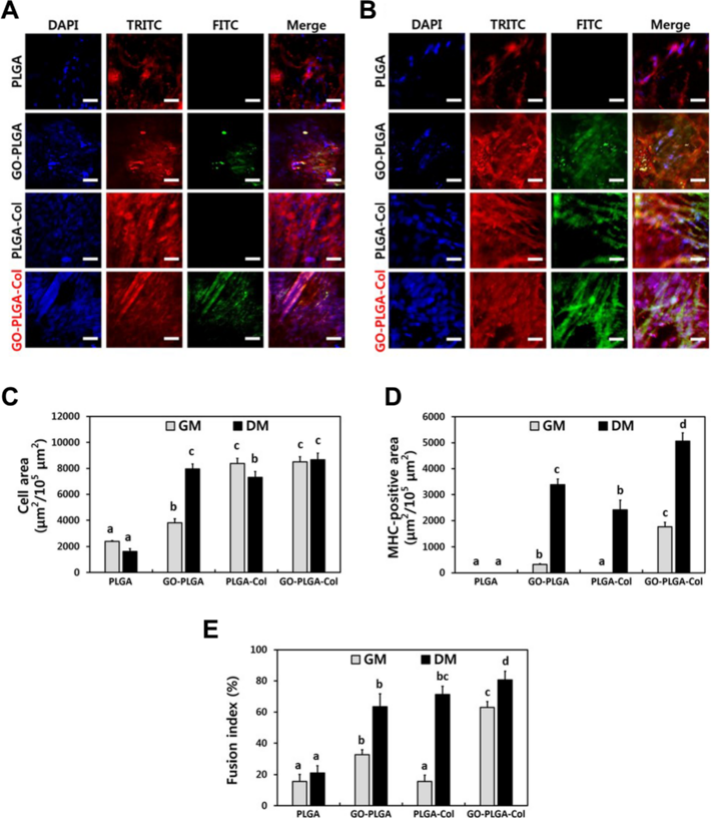
**Myogenic differentiation analysis with immunofluorescence staining.** Two-photon excitation fluorescence images of C2C12 skeletal myoblasts in **(A)** growth media (GM) and **(B)** differentiation media (DM). The cells were cultured in GM for 2 days and then cultured in GM or DM for additional 5 days. The cell nuclei were counterstained with DAPI (blue), the F-actins were stained with TRITC-labelled phalloidin (red) and the myosin heavy chains (MHCs) were stained with FITC-labelled anti-MHC antibody (green). The scale bars are 50 μm. Quantification of **(C)** the cell area, **(D)** MHC-positive area, and **(E)** fusion index. The fusion index was calculated as a percentage of the nuclei number in multinucleate myotubes with more than two nuclei to the total number of nuclei. Quantitative analysis was performed using ImageJ Software. The different letters in **(C)** and **(D)** denote the significant differences between each experimental group, *p* < 0.05. The different letters in **(E)** denote the significant differences between each experimental group, *p* < 0.05. If two groups have the same single letter (a, b, c, etc.), there is no significant difference between them. If a group is marked with a dual letter (eg, bc), it has a significant difference from the control and other groups marked with ‘a’, but does not from another group marked with ‘b’.

The myogenic differentiation of C2C12 skeletal myoblasts on the GO-PLGA-Col matrices was examined by immunofluorescence staining for the myosin heavy chain (MHC), which is a myogenic differentiation marker. C2C12 skeletal myoblasts were seeded on the fibre matrices and incubated in growth media (GM) or differentiation media [DM, Dulbecco’s modified Eagle’s medium (DMEM) containing 2% horse serum and 1% antibiotic-antimycotic solution] for 5 days. After incubation under each condition, expression of MHC and the formation of multinucleate myotubes were analysed by immunofluorescence staining. When C2C12 skeletal myoblasts are fully proliferated, they differentiated with multinucleate myotubes in DM. As shown in Figure 6A, the C2C12 skeletal myoblasts on PLGA matrices attached imperfectly and presented an abnormal morphology. On the PLGA-Col, GO-PLGA-Col matrices, however, C2C12 skeletal myoblasts were well grown and extensively spread out on the matrices. On the other hand, the green fluorescence of MHC was not detected from the C2C12 skeletal myoblasts on the PLGA and PLGA-Col matrices. In contrast, the cells on the GO-PLGA and GO-PLGA-Col matrices exhibited the green fluorescence of MHC. These results indicated that MHC-positive C2C12 skeletal myoblasts on GO-PLGA and GO-PLGA-Col matrices were fused together to form multinucleate myotubes. Meanwhile, as shown in Figure 6B, the C2C12 skeletal myoblasts on all the matrices, except for the PLGA matrices, were differentiated in DM as a positive control. Figure 6C-E and Table 2 show the cell area (μm^2^/10^5^ μm^2^), MHC-positive area (μm^2^/10^5^ μm^2^) and fusion index (%) to quantify the myogenic differentiation of C2C12 skeletal myoblasts. The fusion index was calculated as a percentage of the nuclei number in multinucleate myotubes with more than two nuclei to the total number of nuclei [53]. As shown in Figure 6C, the cell area of the C2C12 skeletal myoblasts cultured on PLGA-Col and GO-PLGA-Col matrices was significantly (*p* < 0.05) increased compared to that on the PLGA and GO-PLGA matrices in GM. This result is in accordance with the cell attachment and proliferation profiles. On the other hand, in DM, the cell area of the C2C12 skeletal myoblasts cultured on GO-PLGA matrices was significantly (*p* < 0.05) increased because the differentiation of cells was effectively facilitated by GO under the differentiation conditions. In addition, the C2C12 skeletal myoblasts cultured on GO-PLGA-Col matrices showed the highest MHC-positive area and fusion index, regardless of the culture condition (Figure 6D and E). This phenomenon can be attributed to the fact that the GO blended within the matrices promotes myogenic differentiation. Many previous studies support this result by showing that graphene and GO can enhance the differentiation of myoblasts, as well as various types of stem cells, such as mesenchymal, neural, embryonic, and induced pluripotent stem cells [45,53-57]. In particular, it has been reported that the GO can stimulate and accelerate the myogenic differentiation of C2C12 myoblasts because the oxygen-containing functional moieties, including hydroxyl, carboxyl and epoxy groups of the GO may increase the adsorption of serum proteins in culture media [53]. Consequently, the GO-PLGA-Col matrices greatly accelerated spontaneous myoblast fusion as well as the myotube maturation of myoblasts cultured in GM because GO can not only to promote differentiation, but also improve the initial attachment and proliferation on the matrices. Some evidence has indicated that an improvement in attachment and proliferation facilitates the differentiation of C2C12 skeletal myoblasts [52,58]. These results indicated that the GO-PLGA-Col matrices are biofunctional scaffolds with the ability to stimulate the differentiation of skeletal myoblasts and enhance the initial attachment and proliferation of skeletal myoblasts. Therefore, it is suggested that GO-PLGA-Col hybrid matrices can be employed as effective biomimetic scaffolds with excellent biocompatibility and biofunctionality.

**Table 2:** **Quantification of the cell area, MHC-positive area and fusion index**

	**Cell area**		**MHC-positive area**		**Fusion index**	
	**(μm** ^**2**^ **/10** ^**5**^ **μm** ^**2**^ **)**		**(μm** ^**2**^ **/10** ^**5**^ **μm** ^**2**^ **)**		**(%)**	
	**GM**	**DM**	**GM**	**DM**	**GM**	**DM**
PLGA	2388.24 ± 62.69	1617.37 ± 217.34	0	0	15.54 ± 4.50	21.01 ± 4.41
GO-PLGA	3807.41 ± 312.47	7965.43 ± 375.90	323.88 ± 26.87	3385.01 ± 214.63	32.68 ± 3.05	63.47 ± 8.22
PLGA-Col	8378.24 ± 396.10	7310.53 ± 444.89	0	2417.42 ± 373.49	15.54 ± 4.14	71.20 ± 5.31
GO-PLGA-Col	8505.3 ± 391.09	8666.40 ± 504.93	1765.99 ± 170.55	5061.09 ± 316.63	62.90 ± 3.85	80.60 ± 5.66

The quantitative data are expressed as the mean ± standard deviation (SD).

## Conclusions

This study was designed to develop biomimetic scaffolds composed of GO-PLGA-Col fibres by electrospinning and to investigate their physicochemical and mechanical properties, biocompatibility, and biofunctionality. We successfully fabricated the GO-PLGA-Col hybrid fibre matrices with a three-dimensional porous structure similar to that of the natural ECM. Our results showed that GO was well dispersed and blended with Col in the hybrid matrices. It was also revealed that the hydrophilicity of the PLGA matrices was significantly increased by blending GO and Col. An improvement in surface hydrophilicity can provide a favourable microenvironment for cell attachment and proliferation. In addition, the GO-PLGA-Col matrices can effectively enhance the initial attachment and proliferation of C2C12 skeletal myoblasts. Furthermore, these hybrid matrices can induce the spontaneous myogenic differentiation of myoblasts owing to the synergistic effect of GO and Col. These results suggest that the GO-PLGA-Col matrices have excellent biocompatibility, as well as suitable physicochemical and mechanical properties for biomimetic scaffolds. In conclusion, GO-PLGA-Col matrices have potential use as biofunctional scaffolds for skeletal tissue engineering and regeneration.

## Methods

### Fabrication of GO-PLGA-Col fibre matrices

GO-PLGA-Col hybrid fibre matrices were fabricated by electrospinning. Briefly, the PLGA resins [PLA:PGA = 75:25 (mol/mol), molecular weight (MW) = 70–110 kDa, Evonik Industries, Essen, Germany] and Col (Darim Tissen, Seoul, Korea) were dissolved in 1, 1, 1, 3, 3, 3-hexafluoroisopropanol (HFIP, Sigma-Aldrich Co., St Louis, MO). The concentrations of PLGA and Col were 200 and 30 mg/ml, respectively. GO was prepared from flake graphite using a modified Hummers’ method [59]. The GO solution in water was prepared by sonicating for 2 hours to distribute the GO evenly throughout the solution and was blended with the PLGA-Col solution. The concentration of GO was 10 mg/ml. The mixture solution of PLGA, Col and GO was then loaded into a syringe (HSW, Tuttlingen, Germany) with a spinneret needle (21-gauge). A voltage of 14 kV was applied using a DC high voltage power supply (NanoNC, Seoul, Korea). A working distance between the needle tip and the collector was 14 cm and the flow rate was 0.2 ml/h. The fabricated GO-PLGA-Col fibres were collected on a steel rotating wheel covered with aluminium foil. After the spinning process, the GO-PLGA-Col fibre matrices were dried overnight under vacuum at room temperature (RT) to remove the residual solvent. Subsequently, the fabricated fibre matrices were cut into 9 mm diameter discs and sterilised by ultraviolet light prior to use.

### Physicochemical and mechanical characterisations of GO-PLGA-Col fibre matrices

The matrices were coated with an ultrathin layer of gold/platinum prior to analysis. The surface morphology of the matrices was observed by FESEM (Hitachi S-4700, Tokyo, Japan) at an accelerating voltage of 5 kV.

The topography of the matrices was characterised by AFM (NX10, Park Systems Co., Suwon, Korea) in air at RT. Imaging was performed in non-contact mode with a Multi 75 silicon scanning probe at a resonant frequency of ~300 kHz. Image analysis was performed using XEI Software (Park Systems Co.).

Compositional analysis of the GO-PLGA-Col fibre matrices was performed by FT-IR spectroscopy. The FT-IR spectra were collected by a FT-IR spectrophotometer (Nicolet 560, Nicolet Co., Madison, WI). All spectra were recorded in absorption mode in the wavelength range of 1000–2200 cm^−1^ with a resolution of 4.0 cm^−1^ and 16-times scanning.

The water contact angles of the matrices were measured by the sessile drop method using a contact angle measurement system (OCA10, Dataphysics, Filderstadt, Germany). A 10 μl sessile drop of distilled water was formed on all samples.

The XRD patterns were measured using the X-ray diffractometer (Empyrean series 2, PANalytical, Almelo, Netherlands) with Cu-K_α_ radiation (λ = 0.154 nm) at 40 kV and 30 mA. The measurements were taken at a scan rate of 2° min^−1^ over the 2θ range of 5–50° at RT.

To examine the mechanical properties of the matrices, the stress–strain curves of the matrices were obtained by a tabletop tensile tester (LRX Plus Series, Ametek Lloyd Instruments Ltd., Fareham, UK) equipped with a 5 kN load cell under a cross-head speed of 10 mm/min. Prior to testing, 4 types of matrices were cut into a rectangular shape, 40 mm in length and 10 mm in width.

### Cell attachment and proliferation assays

C2C12 skeletal myoblasts were purchased from the American Type Culture Collection (Rockville, MD) and routinely maintained in GM, DMEM (Welgene, Daegu, Korea) supplemented with 10% foetal bovine serum (FBS, Welgene) and 1% antibiotic-antimycotic solution (including 10,000 units penicillin, 10 mg streptomycin and 25 μg amphotericin B per ml, Sigma-Aldrich Co.) at 37 °C in a humidified atmosphere containing 5% CO_2_.

The initial attachment and proliferation were measured by using a cell counting kit-8 (CCK-8, Dojindo, Kumamoto, Japan) according to the manufacturer’s instruction. The number of viable cells was directly proportional to the metabolic reaction products obtained in the CCK-8 assay [16]. Briefly, the C2C12 skeletal muscle cells were seeded at a density of 1 × 10^4^ cells/mL on the PLGA, GO-PLGA, PLGA-Col, and GO-PLGA-Col matrices. The cells were incubated with a CCK-8 solution in the last 2 hours of the culture periods for the initial attachment (6 hours) or proliferation (1, 3, 5, and 7 days) at 37°C in the dark. Parallel sets of cells were cultured on the TCPs and the results were regarded as the positive (+) controls. The absorbance was measured at 450 nm using an ELISA reader (SpectraMax 340, Molecular Device Co., Sunnyvale, CA).

### Myogenic differentiation analysis with immunofluorescence staining

C2C12 skeletal myoblasts were seeded at a density of 1 × 10^4^ cells/mL on PLGA, GO-PLGA, PLGA-Col, or GO-PLGA-Col matrices. The cells were cultured in GM for 2 days. The cells were then cultured in GM or DM for an additional 5 days in order to investigate the myogenic differentiation of C2C12 skeletal myoblasts. The media were changed every 24 hours. After culturing under each condition, the cells were fixed with a 3.7% formaldehyde solution (Sigma-Aldrich Co.) for 10 minutes and immersed in 0.1% Triton X-100 (Sigma-Aldrich Co.) for 5 minutes. Subsequently, the cells were blocked with a 2% bovine serum albumin (BSA, GenDEPOT, Barker, TX) solution in Dulbecco’s phosphate-buffered saline (DPBS, Gibco BRL, Rockville, MD) for 30 minutes. The cells were incubated with an Alexa Fluor 488 conjugated anti-MHC monoclonal antibody (clone MF20, 1:200 in 1% BSA solution in DPBS; eBioscienceInc., San Diego, CA) overnight at 4°C, followed by incubation with TRITC-labelled phalloidin (200 units/ml in methanol, 1:40 in 1% BSA solution in DPBS; Molecular Probes, Eugene, OR) for 20 minutes in the dark at RT. The nuclei were counterstained with a DAPI (Sigma-Aldrich Co.) solution in DPBS. The stained cells were imaged using a custom-built two-photon excitation fluorescence microscope, as described elsewhere, and analysed using ImageJ software (National Institutes of Health, Bethesda, MD) [60,61].

### Statistical analysis

All variables were tested in three independent cultures for each experiment, which was repeated twice (n = 6). The quantitative data is expressed as the mean ± standard deviation (SD). The data was tested for the homogeneity of the variances using the test of Levene, prior to statistical analysis. Statistical comparisons were carried out using a one-way analysis of variance (ANOVA; SAS Institute Inc., Cary, NC, USA), followed by a Bonferroni test for multiple comparisons. A value of *p* < 0.05 was considered statistically significant.

## Abbreviations

AFM: Atomic force microscopy
ANOVA: Analysis of variance
BSA: Bovine serum albumin
CCK-8: Cell counting kit-8
Col: Collagen
DAPI: 4’,6-diamidino-2-phenylindole
DC: Direct current
DM: Differentiation media
DMEM: Dulbecco’s modified Eagle’s medium
DPBS: Dulbecco’s phosphate-buffered saline
ECM: Extracellular matrix
ELISA: Enzyme-linked immunosorbent assay
FBS: Foetal bovine serum
FESEM: Field emission scanning electron microscopy
FITC: fluorescein isothiocyanate
FT-IR: Fourier transform-infrared
GM: Growth media
GO: Graphene oxide
GO-PLGA-Col: Graphene oxide-impregnated poly(lactic-co-glycolic acid)-collagen
HFIP: 1, 1, 1, 3, 3, 3-hexafluoroisopropanol
MHC: Myosin heavy chain
MW: Molecular weight
PLGA: Poly(lactic-co-glycolic acid)
RT: Room temperature
SD: Standard deviation
TCP: Tissue culture plastic
TRITC: tetramethylrhodamine isothiocyanate
XRD: X-ray diffraction
